# Short-term ex vivo tissue culture models help study human lung infectionsA review

**DOI:** 10.1097/MD.0000000000032589

**Published:** 2023-01-06

**Authors:** Jing-Yan Xia, Yi-Fei Zeng, Xue-Jie Wu, Feng Xu

**Affiliations:** a Department of Radiation Oncology, Second Affiliated Hospital of Zhejiang University School of Medicine, Hangzhou, Zhejiang, PR China; b Department of Infectious Diseases, Second Affiliated Hospital of Zhejiang University School of Medicine, Hangzhou, Zhejiang, PR China; c Research Center for Life Science and Human Health, Binjiang Institute of Zhejiang University, Hangzhou, China.

**Keywords:** ex vivo tissue culture model, human lung, lung infection

## Abstract

Most studies on human lung infection have been performed using animal models, formalin or other fixed tissues, and in vitro cultures of established cell lines. However, the experimental data and results obtained from these studies may not completely represent the complicated molecular events that take place in intact human lung tissue in vivo. The newly developed ex vivo short-term tissue culture model can mimic the in vivo microenvironment of humans and allow investigations of different cell types that closely interact with each other in intact human lung tissues. Therefore, this kind of model may be a promising tool for future studies of different human lung infections, owing to its special advantages in providing more realistic events that occur in vivo. In this review, we have summarized the preliminary applications of this novel short-term ex vivo tissue culture model, with a particular emphasis on its applications in some common human lung infections.

## 1. Introduction

To date, most results of human lung infections have been obtained from in vitro experiments using animal models, conserved tissues, and established cell lines. Owing to their non-human background, animal experiments may not fully reflect the actual situation of the human body.^[1]^ Thus, significant differences between animal and human immune systems may hinder the transmission of results obtained in animals to human pathophysiology.^[2–4]^ In addition, due to the price and ethical limitations, some susceptible species do not fully represent human infection, or sometimes do not develop the expected symptoms.^[5,6]^ Carefully conserved tissues pretreated by different tissue fixation techniques, such as formalin and Hepes-Glutamic acid buffer mediated Organic solvent Protection Effect based techniques, have been widely applied for immunohistochemical studies to characterize the immunological profiles of lung infection.^[7,8]^ These data provide limited information on the molecular events involved in human lung infection in vivo, owing to the unexpected effects of fixatives on tissues. Results acquired from established cell lines, which comprise only a singular cell type, cannot represent the complex interaction of different components in intact human lung tissues in vivo and reproduce the complex structures and immunological responses in the human respiratory tract completely.^[9,10]^

**Figure 1.**
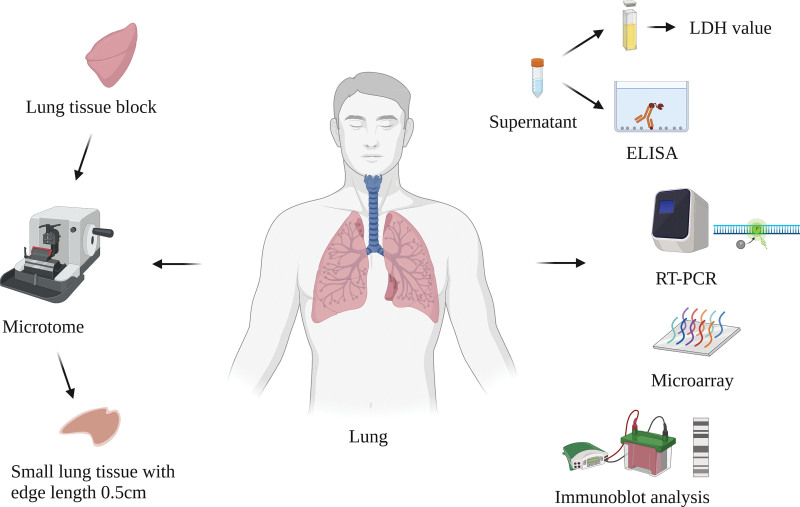
The schematic diagram of ex vivo tissue culture model. Thin lung tissue blocks were prepared using a tissue microtome with edge length approximately 0.5 cm and then were cultivated ex vivo in conditioned cell culture medium. After stimulation/infection with a variety of pathogens in tissue culture, molecular events can be detected ex vivo directly by appropriate techniques. Created with BioRender.com.

The establishment of an ex vivo tissue culture model using fresh and intact specimens may be a possible option for conserving the tissue architecture ex vivo. However, the premise to establish a tissue culture model is capable of producing sufficiently thin tissue sections because the lack of diffusion of oxygen and nutrients in the center of thick tissue cubes may lead to cell death when they are cultured ex vivo. Introducing a tissue microtome solved this problem and enabled the preparation of thin tissue slices.^[11,12]^ Thereafter, a short-term ex vivo tissue culture model was established directly from fresh human lung tissues, which showed some advantages.^[13,14]^ Figure 1 Shows the process of the establishment of an ex vivo tissue culture model.

Although ex vivo short-term tissue culture cannot be propagated, this model provides important information on the complex communication between different tissue cells and the extracellular matrix in intact tissues, and it also overcomes some of the differences in host–defense mechanisms between humans and other animal species.^[15]^ For example, human lung organoid is a newly developed ex vivo tissue culture model. This model can present the in vivo environment and 3D lung structure, and simulate the function of human lung. Additionally, such ex vivo models can represent different part of respiratory tract and offer an opportunity for further studies of the whole respiratory system.^[16]^ This kind of model derived from human pluripotent stem cells which can differentiate to airway and alveolar epithelial cells (AECs) in peripheral blood.^[17,18]^ Human pluripotent stem cells-derived organoids can be induced to differentiate into specific cell types and form tissue-specific organoids.^[19,20]^ Therefore, these cells can be co-cultured with mesenchymal and endothelial cells to build a 3D organoid which represents a part of structure and function of human lung. Although this kind of model may not fully recapitulate the whole functions of mature human lung, it remains an efficient way to study the mechanisms of lung infections because it replicates the human respiratory tract better than previous models.^[21,22]^

This mini-review summarizes the preliminary applications of the above-mentioned novel tissue culture model, with a particular emphasis on its applications in human lung infection.

## 2. Model applications in lung infection

### 2.1. Acute pulmonary *Chlamydia* infection

This ex vivo tissue culture model was first used to investigate acute pulmonary *Chlamydia* infection. Vital lung specimens were found to be infected with *Chlamydia pneumoniae* (*C. pneumoniae*) for at least 48 hours ex vivo. *C. pneumoniae* is predominantly found in alveolar macrophages (AMs). In contrast to acute *Chlamydia* infection, chronic obstructive pulmonary disease (COPD) patients with persistent *Chlamydia* infection showed a significantly higher infection rate in type I AECs, which increased from 2.3 ± 0.9% to 18.2 ± 3.5%. However, only a few bronchial epithelial cells were found to be infected. *Chlamydial* viability and virulence were confirmed by detecting the expression of chlamydial heat shock protein 60 messenger ribonucleic acid in an acute *Chlamydia* infection model.^[23]^ C-X-C motif chemokine ligand (CXCL)-8 expression, an important early innate response mediator, and Toll-like receptor 2 (TLR2), but not Toll-like receptor 4 (TLR4), were significantly increased in the acute *Chlamydia* infection model compared to COPD patients with persistent *Chlamydia* infection. Further studies showed that CXCL8 secretion was reduced dramatically when TLR2 signaling, but not TLR4, was blocked by a neutralizing antibody. Therefore, TLR2 signaling plays an important role in the early innate response caused by acute *Chlamydia* infection.^[24]^

### 2.2. *Streptococcus pneumoniae* (*S. pneumoniae*) infection

Later, application of this ex vivo tissue culture model was extended to explore acute *S. pneumoniae* infection, and similar to *Chlamydia* infection, *S. pneumoniae* was detected predominantly in AM with 80 to 90% positive rate for *S. pneumoniae* deoxyribonucleic acid after 24-hour infection. Depletion of AM with clodronate/liposomes revealed that AM was responsible for cytokine release from lung tissue. Furthermore, lung cell apoptosis has been shown to be associated with marked caspase-3 activation after *pneumococcal* infection in a time-dependent manner. In this study, although both TLR2 and TLR4 were upregulated in response to *pneumococcal* stimulation, they had negligible effects on the expression of interleukin (IL)-8, tumor necrosis factor-α (TNF-α), and IL-6. However, inhibition of p38 mitogen-activated protein kinase (MAPK) signaling markedly reduced the production of inflammatory mediators, suggesting that p38 MAPK signaling may play a crucial role during *S. pneumoniae* infection.^[25]^ Moreover, cyclooxygenase-2 was confirmed to be involved in *S. pneumoniae* infection in this model. Cyclooxygenase-2 was upregulated in AM, vascular endothelium, and alveolar type III epithelial cells, but not type I epithelial cells. Extracellular regulated protein kinase and p38 MAPK signaling were found to be correlated with COX-2 induced prostaglandin E2 formation after *S. pneumoniae* infection. Further functional analysis showed that COX-2 and prostaglandin E regulate pro-inflammatory and anti-inflammatory mediators such as TNF-α, IL-1β, granulocyte-macrophage colony-stimulating factor, platelet-derived growth factor, IL-10, IL-1RA, IL-15, and IL-17 during acute *S. pneumoniae* infection.^[26]^

### 2.3. *Haemophilus influenzae* (*H. influenzae*) infection

Using this tissue culture model, Drömann and colleagues showed that non-typeable *H. influenzae* (NTHi) infection induced a much higher infection rate of AM after acute infection with NTHi-1 and NTHi-2 strains than that of infected COPD lungs. In addition, acute NTHi infection in vitro induced a strong pro-inflammatory response with increased expression of CXCL8, TNF-α, and p38 MAPK, while transforming growth factor-β release decreased. This study also depicted an obvious upregulation of transforming growth factor-pseudoreceptor BMP and activin membrane-bound inhibitor with ubiquitous expression on AM and AEC after in vitro infection of COPD lung tissue, indicating that activin membrane-bound inhibitor might play an important role in the early immune response of pulmonary NTHi infection.^[27]^ In a comparative study, this ex vivo tissue culture model was infected with different respiratory pathogens including *C. pneumoniae*, *S. pneumoniae*, and *H. influenzae*. PCR results showed a significant downregulation of cluster of differentiation 163 (CD163) due to *H. influenzae* and *C. pneumoniae* infections. Further immunohistochemistry confirmed that the expression of pulmonary haptoglobin (pHp) was elevated more frequently in AM than that in AEC II, whereas CD163 expression was downregulated after 24-hour infection with *H. influenzae*. The upregulation of pHp and CD163 caused by *S. pneumoniae* infection was found to be mainly located in AEC II and AM. These results suggested that pHp and CD163 may be functional immunoregulatory elements in human lung infection.^[28]^

### 2.4. *Legionella pneumophila* (*L. pneumophila*) infection

Recently, this ex vivo tissue culture model has been utilized to determine the early stages of *L. pneumophila* infection. It was shown that *L. pneumophila* adhered to the alveolar lining and primarily infected AM and could replicate within this tissue culture model. This study revealed for the first time that *L. pneumophila* outer membrane vesicles, which contain many virulence-related proteins bound predominantly to AM surfaces, could be detected in the cytoplasm. In addition, downregulation of the macrophage receptor with collagenous structure was identified by transcriptome analysis and was further confirmed at the sites of pathogen-infected tissue destruction using immunohistochemistry.^[29]^

### 2.5. *Influenza A virus* infection

Moreover, the ex vivo tissue culture model also assisted in the study of other types of lung infections, such as viral and protozoal infections. Some subtypes of *influenza A virus* can lead to acute severe respiratory disease, resulting in epidemics every year.^[30]^ It has been reported that using this ex vivo culture model can help understand the mechanisms that support or restrict the growth of influenza viruses in the lower respiratory tract. The research found that after 24-hour infection, influenza virus-positive cells were predominantly AEC II. In addition, different kinds of viral antigens could also be detected in AM in infected lung specimens, but they constituted only 4 to 11%. However, although different *influenza A viruses* shared the same cell tropism for AEC II, large differences existed between the strains with regard to replication and cytokine induction, which might explain why different subtypes showed different infectivity and virulence. For example, porcine and low-pathogenicity *avian influenza viruses* were observed to have growth restriction, while the seasonal and pandemic *H1N1 viruses* propagated efficiently. At the same time, *avian influenza viruses* were found to induce a considerably stronger cytokine response in the alveolar tissue than other human-adapted viruses, such as pandemic *H1N1-2009 virus* and the seasonal *H3N2* and *H1N1 viruses*, even in the lack of recruited immune cells. The results showed differential induction of cytokines and chemokines in human lung tissues by human and animal *influenza A viruses*. This might explain why different subtypes grow differently in the human lungs.

These results indicated that differences in the pathogenicity of *influenza A viruses* in the human lung cannot be attributed to different cellular tropisms.^[10]^ Instead, it depended on virus’s inherent replicative properties in AEC II.^[31]^ This research also stressed the value of ex vivo human lung cultures models in pathophysiological processes study.^[10]^

### 2.6. *Cryptosporidium* infection

Generally, *Cryptosporidium* infection causes self-limiting diarrhea in immunocompetent individuals,^[32]^ but it can also target the respiratory tract, causing respiratory cryptosporidiosis in both immunocompetent and immune-deficient individuals.^[33]^ However, the specific pathophysiology of respiratory infections is still unclear. Thus, ex vivo human lung culture models can be used to examine whether the parasite can infect the lungs and finish its life cycle. Within 24 hours after oocyst injection, quantification of 18S ribosomal ribonucleic acid showed that the parasite dramatically increased in the lung model. By performing immunofluorescence assays using a zoite-specific antibody, this model verified the development of asexual (meront I) and (macrogamont) stages. Finally, newly formed oocysts were observed in sporozoite-infected organoids at 6th day post-infection. Therefore, ex vivo human lung culture models allowed *C. parvum* to propagate and complete the full life cycle, which is a potential tool for further research.^[34]^

### 2.7. Coronavirus disease 2019 (COVID-19)

In recent years, the ongoing COVID-19 pandemic, caused by severe acute respiratory syndrome coronavirus 2 (SARS-CoV-2), has created an immense global health crisis.^[35]^ The widely used cell culture infection models that typically use monolayers of a single cell type can support SARS-CoV2 replication and have been successfully applied to SARS-CoV-2 study.^[36]^ For example, these models can help explore cell-autonomous defense mechanisms,^[37]^ host cell interactions,^[38]^ virus replication kinetics^[39]^ and assess the effect of different kinds of medicines.^[40,41]^ However, these simple cell culture models cannot explain the complex pathophysiological processes in organ level, due to the lack of cell-type diversity. Besides, the use of susceptible animal models is also limited because it may lead to problems such as high cost, moral and ethical controversies.^[42]^

An ex vivo human lung culture model has been used to study the mechanisms of SARS-CoV-2 infection.^[43–45]^ The research showed that SARS-CoV-2 replicated in human lung tissues very efficiently within a 48-hour interval. SARS-CoV-2 targeted AEC I, II, and AM primarily in cell tropism. Importantly, SARS-CoV-2 did not significantly lead to type I, II, or III interferons (IFNs) in infected human lung tissues, despite highly efficient virus replication. In addition, SARS-CoV-2 infection only upregulated 38.46% of key inflammatory mediators, including IL-6, monocyte chemoattractant protein-1, CXCL1, CXCL5, and CXCL10 (IP10). Therefore, some untested patients with COVID-19 showed mild or even no symptoms, resulting in spreading the virus in communities and hospitals unknowingly.^[45,46]^ Furthermore, the ex vivo lung culture model will also help understand the earliest stages of SARS-CoV-2 infection in the human respiratory system and reduce the late-phase morbidity and mortality of COVID-19. It was found that lung cells presented a limited innate immune response to SARS-CoV-2, with an obvious lack of type I and III IFNs, contrasting with the strong innate immune response to the influenza virus. This indicated that the early innate immune response resulted in restricted viral clearance once the virus got access to the lung. Moreover, because type III IFN has been proven to regulate critical immune activities,^[47]^ avoiding excessive local inflammation, the obvious lack of type I and III IFNs upregulation in early SARS-CoV-2-infected lung tissues could explain the uncontrolled SARS-CoV-2 replication in late-phase and imbalanced hyperinflammatory response of severe COVID-19. Therefore, studies using this ex vivo tissue culture model can be further employed to assess the impact of viral evolutionary changes and evaluate new therapies against SARS-CoV-2.^[43]^

### 2.8. New techniques in ex vivo lung model conservation

The ex vivo lung model is an efficient tool for human lung infection study and can be implied in other fields as well. However, one challenging problem is to keep the cell viability in this kind of model for a relatively long time. In recent years, the emergence of a new technique named ex vivo lung perfusions (EVLP) can be a solution to this problem. EVLP is regarded as the most effective technique for estimating marginal donor lungs and may help to prolong the normothermic preservation time of ex vivo lung tissues.^[48,49]^ Most importantly, it can potentially recondition impaired lung tissues and restore good state of them.^[50]^ The basic mechanism of EVLP is using a pump to perfuse pulmonary artery with high oncotic pressure preservation fluid which is pure or mixed with red blood cells and recollecting the liquid in a reservoir.^[51]^ At present, there are 3 main EVLP techniques: the Lund, the Organ Care System and the Toronto protocols.^[50]^ Nevertheless, the high cost of EVLP limit its application in science research and clinic.^[49]^ Therefore, it’s necessary to improve current EVLP technology to have longer running time and lower running cost. EVLP can be an effective way to retain viability of the ex vivo lung model in the future.

## 3. Conclusion

In summary, the short-term ex vivo tissue culture model mimics the in vivo tissue microenvironment with a complete tissue architecture and has been applied in different kinds of lung infections (Table 1). This model represents a promising tool for exploring molecular events during acute lung, as it allows the investigation of the early innate immune response in the human lung. However, it also has some inherent limitations: a short period of cell viability; inability of cells to propagate; lack of recruitment of cells and mediators from blood; incomplete compensation by conditioned cell culture medium; bypassing the natural route of bacterial infection; availability of such human lung tissue samples is limited; cannot represent the effect of host systemic inflammatory response and the adaptive immune response; and limited complex interactions with the host.^[25,26,34,45]^ In addition to its application in lung infections, it may also be an important tool for identifying new potential molecular targets for immunotherapy and chemotherapy in lung cancer.^[14,52]^ We believe that this model, in particular, combined with other techniques, will be widely applied in further research beyond human lung infections.^[53]^

**Table 1:** Different applications of the ex vivo tissue culture model.

Application	Major roles
Pulmonary *Chlamydia* infection	Help confirm viability and virulence of *Chlamydial*.
*Streptococcus pneumoniae* infection	Help confirm p38 MAPK signaling and COX2 are involved in *S. pneumoniae* infection.
*Haemophilus influenzae* infection	Help demonstrate NTHi infection induce a much higher infection rate of AM after acute infection with strains NTHi-1 and NTHi-2 than that of infected COPD lungs.
*Legionella pneumophila* infection	Help prove *L. pneumophila* infects AM primarily.
*Influenza A virus* infection	Help understand the mechanisms that support or restrict the growth of influenza viruses in the lower respiratory tract.
*Cryptosporidium* infection	Help examine whether the parasite can infect the human lung and finish its life cycle.
Coronavirus disease 2019	Help study the mechanisms of SARS-CoV-2 infection.

AM = alveolar macrophage, COPD = chronic obstructive pulmonary disease, COX2 = cyclooxygenase-2, *L. pneumophila* = *Legionella pneumophila*, MAPK = mitogen-activated protein kinase, NTHi = nontypeable *Haemophilus influenzae*, SARS-CoV-2 = severe acute respiratory syndrome coronavirus 2.

## Author contributions

J-YX conceived and participated in drafting the manuscript and gave the final approval of the version to be submitted and of any revised version. Y-FZ was responsible for writing the first draft, and was involved in the final approval of the version to be published. X-JW was responsible for collecting the documents. FX critically revised the manuscript for important intellectual content.

**Supervision:** Xue-Jie Wu, Feng Xu.

**Writing – original draft:** Jing-Yan Xia, Yi-Fei Zeng, Feng Xu.

**Writing – review & editing:** Yi-Fei Zeng, Xue-Jie Wu.
